# Melanoma Proteomics Unveiled: Harmonizing Diverse Data Sets for Biomarker Discovery and Clinical Insights via MEL-PLOT

**DOI:** 10.1021/acs.jproteome.4c00749

**Published:** 2025-05-05

**Authors:** Áron Bartha, Boglárka Weltz, Lazaro Hiram Betancourt, Jeovanis Gil, Natália Pinto de Almeida, Giampaolo Bianchini, Beáta Szeitz, Leticia Szadai, Indira Pla, Lajos V. Kemény, Ágnes Judit Jánosi, Runyu Hong, Ahmad Rajeh, Fábio Nogueira, Viktória Doma, Nicole Woldmar, Jéssica Guedes, Zsuzsanna Újfaludi, Yonghyo Kim, Tibor Szarvas, Zoltan Pahi, Tibor Pankotai, A. Marcell Szasz, Aniel Sanchez, Bo Baldetorp, József Tímár, István Balázs Németh, Sarolta Kárpáti, Roger Appelqvist, Gilberto Barbosa Domont, Krzysztof Pawlowski, Elisabet Wieslander, Johan Malm, David Fenyo, Peter Horvatovich, György Marko-Varga, Balázs Győrffy

**Affiliations:** 1 Department of Bioinformatics, 37637 Semmelweis University, Budapest 1085, Hungary; 2 Department of Pediatrics, 37637 Semmelweis University, Budapest 1085, Hungary; 3 Cancer Biomarker Research Group, Institute of Molecular Life Sciences, 280964 Research Centre for Natural Sciences, H-1117, Budapest, Hungary; 4 European Cancer Moonshot Lund Center, Lund, SE-221 84, Sweden; 5 Clinical Protein Science & Imaging, Biomedical Centre, Department of Biomedical Engineering, 5193 Lund University, Lund, 223 63, Sweden; 6 9372 San Raffaele Hospital, Milan, 20132, Italy; 7 Division of Oncology, Department of Internal Medicine and Oncology, 37637 Semmelweis University, Budapest, 1085, Hungary; 8 Department of Dermatology and Allergology, 37442 University of Szeged, Szeged, 6720, Hungary; 9 Department of Biomedical Engineering, Faculty of Engineering, LTH, 5193 Lund University, Lund, 22363, Sweden; 10 HCEMM-SU Translational Dermatology Research Group, 37637 Semmelweis University, Budapest, 1085, Hungary; 11 Department of Physiology, 37637 Semmelweis University, Budapest, 1094, Hungary; 12 Department of Dermatology, Venerology and Dermatooncology, Faculty of Medicine, 37637 Semmelweis University, Budapest, 1085, Hungary; 13 Institute for Systems Genetics, 12296 NYU Grossman School of Medicine, New York, New York 10016, United States; 14 Department of Biochemistry and Molecular Pharmacology, 12296 NYU Grossman School of Medicine, New York, New York 10016, United States; 15 Department of Dermatology, 2348 Massachusetts General Hospital, Harvard Medical School, Boston, Massachusetts 02114, United States; 16 Proteomics Unit, Institute of Chemistry and Research Center for Precision Medicine, Institute of Biophysics Carlos Chagas Filho, Federal Univesity of Rio de Janeiro, Rio de Janeiro, 21941-170, Brazil; 17 Chemistry Institute Federal, 28125 University of Rio de Janeiro, Rio de Janiero, 21941-909, Brazil; 18 37442 University of Szeged, Albert Szent-Györgyi Clinical Centre, Department of Pathology, 6720, Szeged, Hungary; 19 Drug Discovery Platform Research Center, Therapeutics and Biotechnology Division, 65680 Korea Research Institute of Chemical Technology, Daejeon, 34114, Republic of Korea; 20 Department of Urology, 37637 Semmelweis University, Budapest, 1082, Hungary; 21 Department of Urology, University of Duisburg-Essen, Essen, 45147, Germany; 22 Department of Pathology, Albert Szent-Györgyi Medical School, 37442 University of Szeged, Állomás utca 1, Szeged H-6725, Hungary; 23 Hungarian Centre of Excellence for Molecular Medicine (HCEMM), Genome Integrity and DNA Repair Core Group, 37442 University of Szeged, Budapesti út 9, Szeged H-6728, Hungary; 24 Competence Centre of the Life Sciences Cluster of the Centre of Excellence for Interdisciplinary Research, Development and Innovation, 37442 University of Szeged, Dugonics tér 13, Szeged H-6720, Hungary; 25 Division of Oncology, Department of Internal Medicine and Oncology, 37637 Semmelweis University, 1085 Budapest, Hungary; 26 Section for Clinical Chemistry, Department of Translational Medicine, Lund University, Skåne University Hospital Malmö, Malmö,205 02, Sweden; 27 Division of Oncology, Department of Clinical Sciences Lund, 5193 Lund University, Lund,221 84, Sweden; 28 Department of Pathology, Forensic and Insurance Medicine, Faculty of Medicine, 37637 Semmelweis University, Budapest,1085, Hungary; 29 Department of Biochemistry and Microbiology, Warsaw University of Life Sciences, Warszawa,02-787, Poland; 30 Department of Molecular Biology, 12334 University of Texas Southwestern Medical Center, Dallas, Texas 75390-9148, United States; 31 Section for Clinical Chemistry, Department of Translational Medicine, 5193 Lund University, Lund 21428, Sweden; 32 University of Groningen, Groningen Research Institute of Pharmacy, Analytical Biochemistry, Groningen, 9711, The Netherlands; 33 Dept. of Biophysics, Medical School, University of Pecs, H-7624, Pecs, Hungary

**Keywords:** mass spectrometry, survival, proteomics, tumor progression, skin cancer

## Abstract

Using several melanoma proteomics data sets we created a single analysis platform that enables the discovery, knowledge build, and validation of diagnostic, predictive, and prognostic biomarkers at the protein level. Quantitative mass-spectrometry-based proteomic data was obtained from five independent cohorts, including 489 tissue samples from 394 patients with accompanying clinical metadata. We established an interactive R-based web platform that enables the comparison of protein levels across diverse cohorts, and supports correlation analysis between proteins and clinical metadata including survival outcomes. By comparing differential protein levels between metastatic, primary tumor, and nonmalignant samples in two of the cohorts, we identified 274 proteins showing significant differences among the sample types. Further analysis of these 274 proteins in lymph node metastatic samples from a third cohort revealed that 45 proteins exhibited a significant effect on patient survival. The three most significant proteins were HP (HR = 4.67, p = 2.8e-06), LGALS7 (HR = 3.83, p = 2.9e-05), and UBQLN1 (HR = 3.2, p = 4.8e-05). The user-friendly interactive web platform, accessible at https://www.tnmplot.com/melanoma, provides an interactive interface for the analysis of proteomic and clinical data. The MEL-PLOT platform, through its interactive capabilities, streamlines the creation of a comprehensive knowledge base, empowering hypothesis formulation and diligent monitoring of the most recent advancements in the domains of biomedical research and drug development.

## Graphical abstract

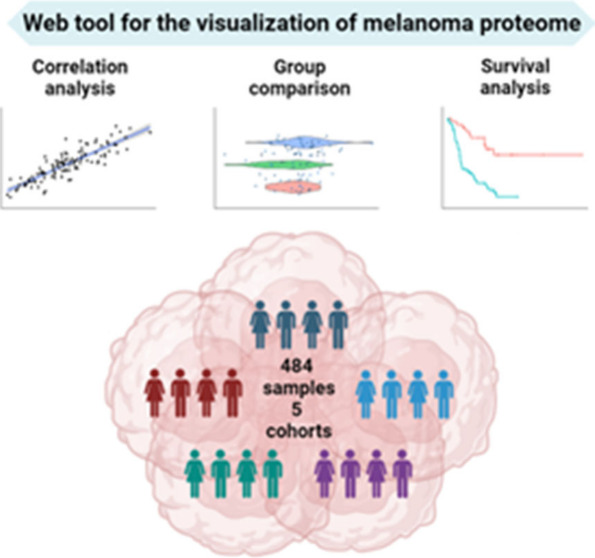

## Introduction

Proteins are the primary actors in biological molecular events, and they play a crucial role as biomarkers for diagnosing and estimating the prognosis of diseases and common targets for therapeutic interventions. Presently, a majority of drugs are designed to target proteins, and mass spectrometry (MS)-based proteomics has proven to be a valuable analytical method in drug discovery.^1^ The simultaneous analysis of a large number of proteins in a complex clinical sample is termed proteomics, which can provide deeper insights into changes, interactions, and functions of proteins related to a particular disease. Furthermore, this approach has the potential to unveil the functional state of different subgroups of samples or individuals, leveraging cutting-edge high-throughput profiling analytical platforms.

Melanoma, one of the most prevalent cancers among those under the age of 40, exhibits immense genetic variation.^2^ Mutations affecting driver genes can disrupt cell cycle control, DNA repair, and signaling pathways. The BRAF V600E mutation, the most frequently observed driver mutation in melanoma, is found in about half of all cases. It activates MAPK signaling pathways, influencing cell proliferation and differentiation.^3^ The well-known heterogeneity of melanoma is a known contributor to treatment resistance and disease progression.

Previously, we have established web services enabling the discovery and validation of biomarker candidates for predicting therapy response,^4^ assessing survival,^5^ understanding the effects of genetic mutations,^6^ and analyzing metastases^7^ across various cancer types. A major advantage of these web-based analysis portals is that users can address specific biological questions without requiring extensive bioinformatics expertise. However, these tools have primarily focused on genomic and transcriptomic data. In contrast, proteins are directly responsible for biological functions and offer stronger connections to clinical features, including patient outcomes. Studying functional protein expression in cancer is essential for better understanding the disease and improving patient care. These studies help reveal the molecular mechanisms behind cancer progression and offer many benefits, such as understanding tumor biology, finding biomarkers, developing new drugs, and personalizing treatments.

Proteomics has recently emerged as a significant field in cancer research, with the CPTAC project leading to comprehensive examinations of various malignancies, including breast cancer,^8^ lung adenocarcinoma,^8^ endometrial carcinoma,^9^ and several others.^10^ These studies have generated vast amounts of multiomic data that are invaluable to the research community. Online platforms like LinkedOmics^11^ and UALCAN^12^ have integrated multiomic data from the TCGA and CPTAC projects, providing relatively easy access to these data resources.

In the field of melanoma research, tools that incorporate mass spectrometry (MS)-based proteomic data are still limited, even though RPPA-based data sets are available and widely used.^13^ Recent advancements in multiomic and proteomic studies have greatly enhanced our understanding of melanoma biology, leading to the discovery of novel prognostic biomarkers and the identification of proteome-based subtypes.^14^ Additionally, proteomic profiling of metastatic melanoma has highlighted site-specific cellular processes and potential therapeutic responses.^15^ Although the aforementioned studies include a substantial amount of protein-based data, these data are not readily accessible through user-friendly platforms. Furthermore, proteome-based melanoma data sets are notably absent, in contrast to the extensive data sets available for breast and colon cancers through CPTAC.

Here, our aim was to develop a unique tool for discovering protein-based biomarkers in melanoma research, and make a user-friendly webtool to mine the data of our previously published melanoma studies.^16–21^ We assembled data from five melanoma cohorts including both normal, primary tumor, and metastatic tissues and then constructed a user-friendly web portal equipped with built-in statistical and visualization capabilities. By the utilization of the created platform and analysis pipeline, we successfully identified a set of candidate biomarker proteins that may predict progression. Consequently, MEL-PLOT provides a toolbox solution for functional protein expression studies in cancer that are invaluable for advancing both our scientific understanding and clinical management of melanoma.

## Methods

### Cohorts Included in the Analysis

Proteomic data was collected from our previously described publications.^16–21^ The database is comprised of five sample cohorts referred to as Cero (FFPE samples),^16^ Primero,^17^ Segundo,^18^ Tercero,^19^ and Cuarto^19^ (all four contains fresh frozen samples). All patients provided informed consent, and each study received approval from local and regional ethical committees. The ethical approval numbers for the Cero, Primero and Segundo, Tercero, and Cuarto studies were MEL-PROTEO-001 (University of Szeged, Hungary), DNR 191/2007, 2013/339, 2013/101 (Lund University, Sweden), TUKEB 114/2012 (Semmelweis University, Hungary), and 191–4/2014 (Semmelweis University, Hungary), respectively. All samples are recoded, with specific identification numbers in order to avoid patient identification.

The Cero study consists of 90 FFPE samples obtained from primary tumors, lymph nodes, and cutaneous metastases of 77 patients (38 men and 39 women) diagnosed with melanoma between 2005 and 2020. Dominant histological subtypes of primary tumors include superficial spreading melanoma (SSM) with vertical growth and nodular melanoma (NM). The mean age ± standard deviation (range) at primary diagnosis was 64.3 ± 10.9 (31–91) years. Disease-free survival from primary to metastasis was 1.4 ± 2.3 (0–12.0) years, and overall survival was 4.3 ± 3.7 (0–17.0) years. The cohort comprises 36 patients with wild-type BRAF status and 38 patients with the V600E mutation in the BRAF gene (one patient had D587G mutation). Patients were treatment-naive at sample collection, with 18 patients receiving no treatment after sample collection. Others underwent multiple treatment cycles including immunotherapy, targeted therapy, and other interventions (radiation therapy, chemotherapy, interferon therapy, and electrochemotherapy).

The Primero study includes lymph node metastasis samples from 111 patients (68 men and 43 women) diagnosed with stage 3 or 4 metastatic melanoma between 1975 and 2011. Dominant histological subtypes of primary tumors were SSM and NM. Average age ± standard deviation (range) at lymph node metastasis diagnosis was 62.4 ± 13.7 (25–89) years. Time elapsed to progression from primary tumor to lymph node metastasis was 5.0 ± 5.6 (0–18.0) years, and overall survival was 7.9 ± 6.8 (0.2–43.0) years. The cohort includes 59% of patients with wild-type BRAF status and 36% with the V600E mutation in the BRAF gene (4% had V600A or V600 K mutation).

The Segundo study comprises 137 patients diagnosed with metastatic melanoma between 1975 and 2011. All samples were collected before patient treatment. Notably, there is an overlap between samples in the Primero and Secundo study, but the used analytical method is different. The average age ± standard deviation (range) at metastasis diagnosis was 62.3 ± 13.7 (25–89) years, and the cohort predominantly consisted of males (65%). Among patients with available overall survival (OS) information, 50% survived less than five years. Samples included fresh frozen metastatic tissues from lymph nodes (n = 126), subcutaneous (n = 7), cutaneous (n = 1), and visceral (n = 3) metastases. The origin was unknown for five samples. Fifty tumors had the BRAF (V600E) mutation, 36 had NRAS Q61K/R mutation, and 37 tumors had wild-type variants for both genes.

In the Tercero study, 74 fresh-frozen samples of distant metastases were collected after the patient’s death from 22 patients (15 men and 7 women) diagnosed with melanoma. The number of metastases per patient ranged from one to nine. Metastases originated from various tissues, such as lung (n = 14), liver (n = 11), adrenal (n = 6), spleen (n = 6), and kidney (n = 5). Age at diagnosis of the primary tumor was 52.6 ± 14.5 (32–78) years. Overall survival was 4.3 ± 3.2 (0.7–11.4) years. Similar to previous studies, samples were classified either SSM or NM. The cohort included seven patients with wild-type BRAF status, 14 with BRAF V600E, one with BRAF K601E, and two with BRAF V600 K (all checked in the corresponding primary tumor). Among patients with WT BRAF, three had an NRAS mutation (one Q61K and two Q61R). Most patients received treatment before sample collection, including IFN, chemotherapy, and targeted therapy (vemurafenib). Three patients did not receive any treatment, and information about treatment history was unavailable for four patients.

The Cuarto cohort of samples is derived from a prospective melanoma study involving 77 surgically resected tissue samples from 47 melanoma patients. Study participants had an average age of 65 years at diagnosis, with 27 being male, accounting for 57% of the total. The samples were stored as fresh-frozen and prepared for quantitative proteomics using a previously described method.^22^

### Determination of Protein Levels

In the Cero study, as presented in prior work, identification and label-free quantification were conducted using a Q-Exactive HF-X (Orbitrap analyzer) platform in DDA mode.^16^ The database search was performed using Proteome Discoverer 2.4, employing the UniProt human database (2020/05/26) and two spectral libraries, namely the Proteome tools HCD 28 PD and NIST Human Orbitrap HCD. Raw protein intensities were log2-transformed and median-normalized by centering them around the global median, calculated using all nonzero values in the data set as described previously.^16^

For mass spectrometry analysis in the Primero study, a Q-Exactive Plus (Orbitrap analyzer) was utilized in DDA mode, and label-free quantification was employed.^17^ The database search was executed using Proteome Discoverer, using a UniProtKB database version from May 2016, excluding isoforms. Log2-transformed protein intensity values were normalized by subtracting the sample median, as previously reported.^17^

In the Segundo study, MS was performed using a Q Exactive HF-X (Orbitrap analyzer) in DDA mode, and quantification was achieved through TMT isobaric tags (11 plex). Database search utilized the Proteome Discoverer software, with the database sourced from UniProtKB (2018.10.01). Peptides uniquely mapped to a protein were used to estimate relative protein abundances. To correct for experimental differences, including sample handling variability and biases such as column changes, protein intensities were log2-transformed and normalized by centering them around zero (achieved by subtracting the median intensity of each sample). To ensure comparability of relative protein abundances across different TMT11-plex batches, the intensities from the pooled reference sample (channel 126 in each batch) were subtracted from the intensities of each channel within the corresponding batch. This adjustment provided the final relative protein abundance values, as previously described in.^18^

MS measurements for the Tercero project employed a Q-Exactive HF-X (Orbitrap analyzer) in DIA mode, with label-free quantification. Protein and peptide identification and quantification were performed using Spectronaut software (Biognosys, AG). DDA raw files from individual samples and pooled samples, processed with the same workflow, were searched together against a human protein database downloaded from UniProt in 2018/10/01 to create a spectral library. The search engine used was Pulsar, which is integrated into the Spectronaut platform as described previously.^19^ Log2-transformed protein intensities underwent median scale normalization and were centered around the global median. The study identified 10,122 different proteins.

MS data for the Cuarto cohort was acquired using Q-Exactive HF-X, following a high-resolution DIA-MS approach. A custom spectral library was employed to search for proteins in the samples, utilizing the Spectronaut search engine. Protein and peptide identification and quantification were performed using Spectronaut software (Biognosys, AG). DDA raw files from individual and pooled samples, processed using the same workflow, were searched together against a human protein database downloaded from UniProt in 2018 to create a spectral library. The search engine used was Pulsar, which is integrated into the Spectronaut platform, as previously described.^19^ A total of 9,040 proteins were confidently identified and quantified using a label-free approach. The protein quantification output was log2-transformed and normalized by subtracting the median of each run, as previously reported.^19^

### Web-Platform Setup

In the web platform, both the backend and frontend of the web application have been developed using the R Shiny package (version 1.7.4) and the shinycssloaders and shinymanager additional packages. The ggplot2 package (version 3.4.1) was used to generate the plots presented in all the analysis options. The analyses were conducted using the R statistical programming language (version 4.2.2). Survival analysis was executed with the survival and survminer R packages. To determine the optimal cutoff value for each protein, an assessment was performed across all possible cutoff values, iterating between the lower and upper quartiles of the intensity values. For risk assessment among different cohorts, Cox proportional hazard regression analysis was employed. To examine the combined behavior of two variables, Pearson and Spearman correlation tests were performed using the ’cor’ function from the stats package in R (version 4.2.2). Table of content graphic has been created with BioRender.com. In the case of Exploratory analysis proteins were grouped together based on their UNiProt ID. Proteins with available isoform data were treated separately. If both canonical and isoform identifier were available, those are treated separately. ()

### Data Analysis for the Potential Biomarkers

The Kruskal–Wallis test, followed by Dunn’s posthoc test, was used to assess statistical significance in differential protein expression analysis across primary tumors, lymph node metastases, and cutaneous metastases in the Cero study. Similarly, differential protein expression analysis was performed in the Cuarto study, comparing normal adjacent tissue, primary tumors, and lymph node metastases. The Kruskal–Wallis test was conducted using the inbuilt R package, while Dunn’s posthoc test utilized the ’dunn.test’ R package. To account for multiple hypothesis testing, we adjusted the p-values using the Benjamini-Hochberg method. Effect size for the Kruskal–Wallis test was estimated using eta-squared with the ’rstatix’ package (version 0.7.2). Proteins exhibiting significant differential expression with at least a moderate effect size were selected for further survival analysis in the Segundo cohort, which can be replicated using the survival analysis tool available in the Segundo cohort.

Gene Ontology (GO) analysis was conducted using the clusterProfiler package (version 4.10.0).^23^ Enrichment analysis was performed with the enrichGO function, applying the default settings. The org.Hs.eg.db annotation database was used for the analysis, with all annotated genes in the database serving as the background. To generate the tree plot shown in Figure 2, the treePlot function was used with default parameters. Gene sets with high similarity were grouped using k-means clustering.

For heatmap creation, the R package ComplexHeatmap (version 2.18.0) was used, utilizing the Heatmap function with default parameters.^24^ Log2 intensity values were row-wise scaled using the basic R function “scale”. Hierarchical clustering was applied to perform clustering of the data both column-wise and row-wise.

## Results

### Clinical Data Sets Summary

Our database encompasses a total of 489 tissue samples from five distinct studies, utilizing clinical data from 394 patients. The “Cero study” contains expression data for 7,880 proteins (with 6.42% of missing values), the “Primero cohort” comprises expression data for 4,892 proteins (with 61.74% missing values). Within the “Segundo cohort”, expression data for 12,690 proteins is available (with 15.41% missing values), while the “Tercero cohort” presents expression data for a total of 10,121 proteins (with 22.98% missing values). Expression data for 9,040 proteins (with 26.74% missing values) is accessible within the “Cuarto cohort”. Across all studies, a total of 4,223 proteins are found to overlap. Aggregate characteristics of each data set is presented in Table 1.

**Table 1:** Characteristics of the Melanoma Cohorts Included in the Analysis^a^

Cohort	Patient n	Sample n	Sex female–male	Source	Sample origin	Proteins identified
Cero*	77	90	39–38	FFPE	PT, LN, LR	7,880
Primero	111	111	43–68	FF	LN	4,892
Segundo*	137	137	47–87	FF	LN, CM, DM, PT	12,690
Tercero**	22	74	7–15	FF	DM	10,121
Cuarto	47	77	20–27	FF	NT, TM, PT, LR, CM, LN, DM	9,040

a Abbreviations: Non-tumor (NT), tumor microenvironment (TM), primary tumor (PT), local recurrences (LR), cutaneous metastases (CM), regional lymph node metastases (LN), and distant metastases (DM), Formalin-Fixed Paraffin-Embedded (FFPE), fresh-frozen (FF). *Studies containing survival data. **Samples in the Tercero study were obtained post-mortem.

### Web Platform Overview

Our novel implementation utilizes proteomic data from multiple patient cohorts, providing a more comprehensive view of the molecular landscape of melanoma. The adapted platform features a total of five specialized modules, each tailored to accommodate the unique analytical needs and data complexities associated with the different proteomic cohorts. These modules incorporate several algorithms for a variety of statistical analyses and visualization tools, effectively enabling robust biomarker discovery. Additionally, we introduced a sixth module that serves as an experimental sandbox, specifically designed for exploratory analyses. Within the cohort-specific pages, users can readily access an array of functionalities such as group comparison, correlation analysis, and survival analysis options. Notably, the survival analysis feature is exclusively accessible for cohorts where relevant follow-up data is accessible. For convenient access to this comprehensive analysis web interface, please visit: https://www.tnmplot.com/melanoma.

### Identification of a Progression-Related Protein Panel for Melanoma

Our web-based platform demonstrated its flexibility and analytical capabilities by focusing on an essential research question: understanding the molecular mechanisms that drive melanoma progression. Given the frequent occurrence of metastasis in melanoma, we aimed to identify potential protein-based biomarkers using our established database. Initially, we conducted a differential protein expression analysis in the Cero study, comparing primary tumors, lymph node metastases, and cutaneous metastases. Simultaneously, a similar analysis was performed in the Cuarto study, comparing normal adjacent tissue, primary tumors, and lymph node metastases. Across both studies, 274 proteins exhibited significant differential expression, with effect sizes ranging from 0.11 to 0.46 in the Cero study and 0.1 to 0.73 in the Cuarto study, indicating moderate to large effects. (Figure 1). Global results are summarized in . A Gene Ontology (GO) enrichment analysis provided insights into the functional roles of these proteins. The analysis highlighted prominent GO terms associated with epidermal cell development differentiation, and cytoskeletal organization, emphasizing the importance of these cellular processes in melanoma progression. (Figure 2).

**Figure 1:**
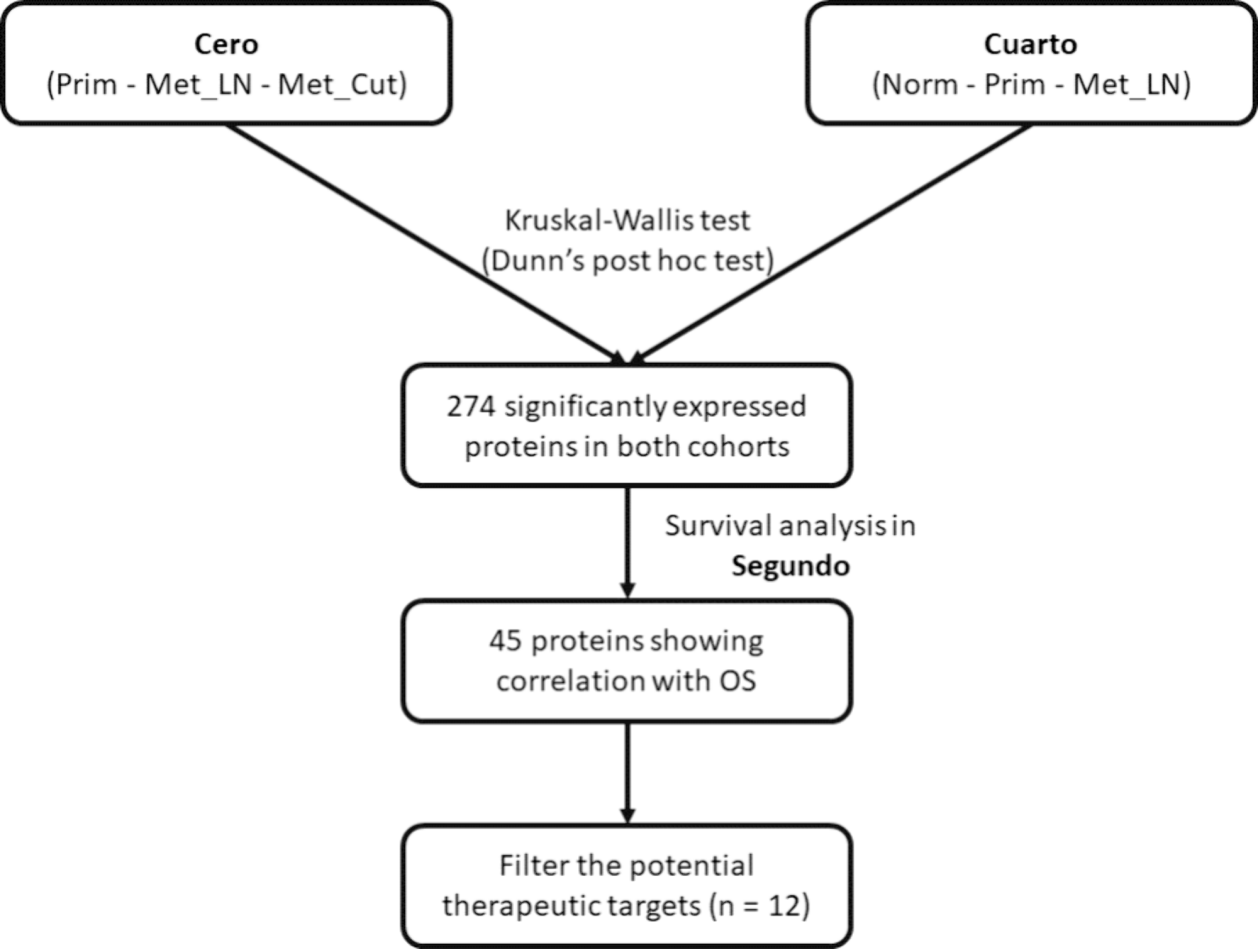
Data analysis workflow Abbreviations: Normal (Norm), Primary tumor (Prim), Lymph node metastasis (Met_LN), Cutaneous metastasis (Met:Cut), overall survival (OS).

**Figure 2:**
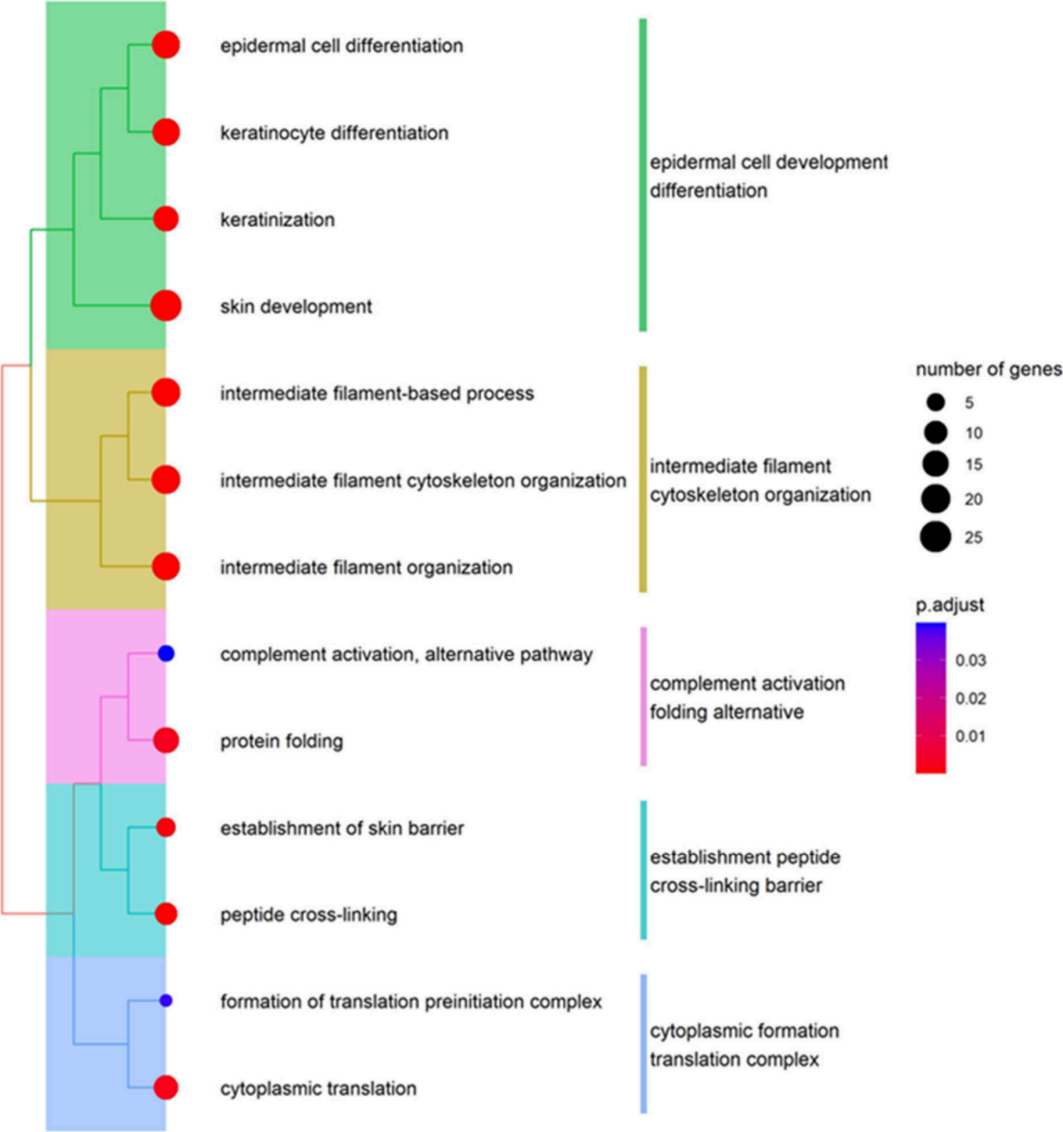
Enriched GO biological process terms of the differentially expressed 274 proteins between localized and metastatic samples.

In the next phase, we focused on the subset of 274 proteins using the Segundo data set, which includes lymph node metastatic samples along with clinical follow-up data. Our goal was to explore how these proteins impact patient survival outcomes when measured in lymph node metastasis which is a common metastatic site of melanoma. Of the 274 proteins, 45 showed a significant association with survival. These 45 proteins were divided into two groups based on their hazard ratios (HR): 12 proteins had an HR greater than 2, indicating a higher risk, while 33 proteins had an HR below 0.5, suggesting a potential protective effect. Elevated expression of 12 proteins among the 45 identified was significantly associated with poorer patient survival outcomes. Among these, Haptoglobin (HP, HR = 4.67, p = 2.8e-06), Galectin-7 (LGALS7, HR = 3.83, p = 2.9e-05), and Ubiquilin-1 (UBQLN1, HR = 3.2, p = 4.8e-05) emerged as the most interesting proteins with a considerable impact among the examined proteins. (Figure 3). A comprehensive list containing all 12 proteins, coupled with their corresponding significance values and effect sizes, is provided in Table 2. As an example of how our web platform can be utilized, we examined the differential protein expression profile of one of the identified proteins (Figure 4).

**Figure 3:**
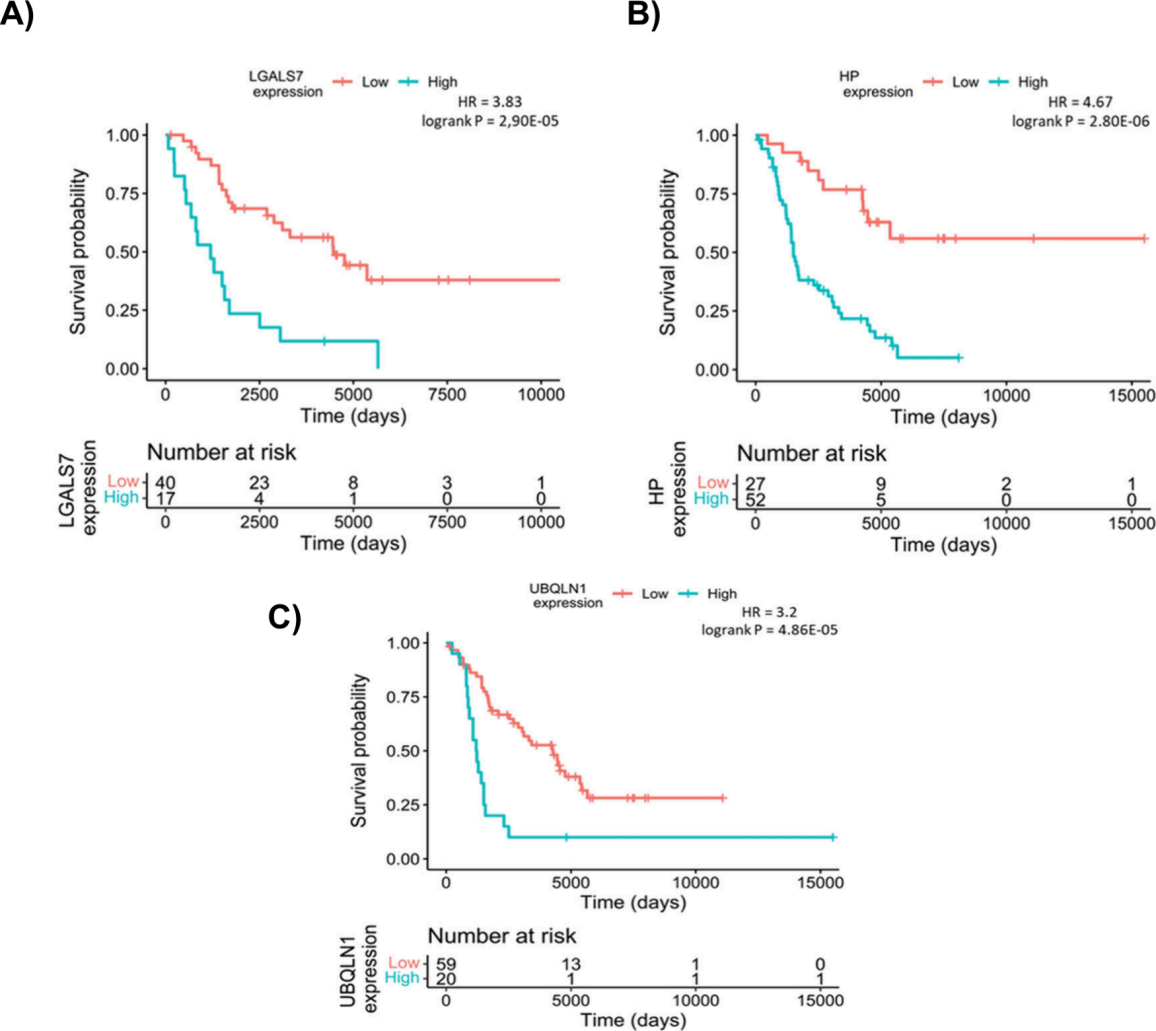
Survival analysis of the top three proteins with higher expression associated to worse survival outcome including LGALS7 (A), HP (B), and UBQLN1 (C).

**Figure 4:**
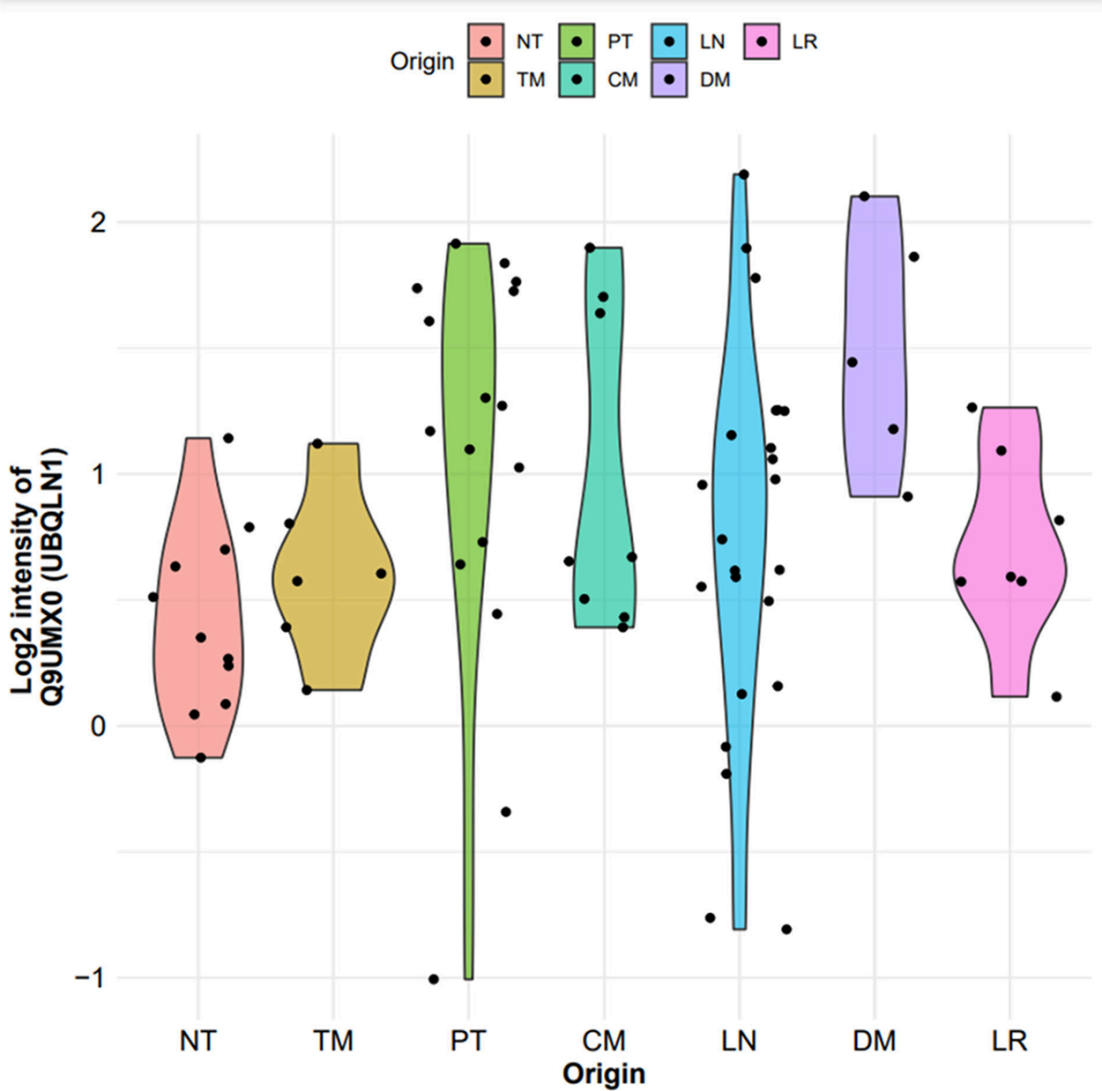
Differential protein expression representation of UBQLN1 using our webtool in the Cuarto data set. Abbreviations: Nontumor (NT), tumor microenvironment (TM), primary tumor (PT), local recurrences (LR), cutaneous metastases (CM), regional lymph node metastases (LN), and distant metastases (DM).

**Table 2:** Top Proteins Showing Differential Expression between Primary Tumor and Lymph Node Metastasis in Both Cero and Cuarto Data Sets^a^

Protein Name	UNIPROT ID	HR	Survival P	K–W P.adj in Cero	Dunn’s P in Cero	Effect Size in Cero	FC in Cero	K–W P.adj in Cuarto	Dunn’s P in Cuarto	Effect Size in Cuarto	FC in Cuarto
APCS	P02743	2.55	5.45 × 10^–04^	1.33 × 10^–03^	1.03 × 10^–06^	0.25	–1.11	1.19 × 10^–04^	2.72 × 10^–02^	0.54	–1.92
CTSG	P08311	3.08	3.27 × 10^–05^	3.91 × 10^–03^	6.42 × 10^–06^	0.21	–0.81	4.42 × 10^–04^	3.21 × 10^–03^	0.35	–2.43
GSN	P06396	2.33	2.21 × 10^–03^	4.32 × 10^–02^	3.21 × 10^–04^	0.12	–0.32	2.79 × 10^–03^	2.72 × 10^–03^	0.25	–0.64
HP	P00738	4.67	2.80 × 10^–06^	1.34 × 10^–02^	2.13 × 10^–04^	0.16	–0.82	4.12 × 10^–02^	2,53 × 10^–01^	0.11	–0.51
ITIH1	P19827	2.1	6.59 × 10^–03^	2.86 × 10^–02^	1.67 × 10^–04^	0.14	0.34	3.79 × 10^–04^	2.70 × 10^–02^	0.36	–0.77
KRT5	P13647	2.28	2.79 × 10^–03^	6.25 × 10^–06^	1.11 × 10^–10^	0.47	–2.02	2.54 × 10^–05^	1.94 × 10^–04^	0.71	–5.53
KRT6A	P02538	2.15	5.54 × 10^–03^	6.25 × 10^–06^	4.01 × 10^–10^	0.45	–2.1	8.10 × 10^–05^	5.59 × 10^–07^	0.58	–6.06
KRT7	P08729	2.59	8.02 × 10^–03^	5.83 × 10^–04^	3.41 × 10^–07^	0.28	–0.92	1.30 × 10^–04^	8.50 × 10^–03^	0.47	–0.61
LGALS7	P47929	3.83	2.90 × 10^–05^	6.25 × 10^–06^	2.08 × 10^–10^	0.45	–2.11	2.54 × 10^–05^	2.61 × 10^–04^	0.73	–6.13
NCCRP1	Q6ZVX7	3.12	9.81 × 10^–05^	3.48 × 10^–03^	6.12 × 10^–06^	0.21	–0.67	1.71 × 10^–04^	1.05 × 10^–03^	0.42	–4.83
SBSN	Q6UWP8	2.52	9.87 × 10^–04^	9.36 × 10^–05^	2.49 × 10^–08^	0.34	–1.19	1.36 × 10^–04^	1.94 × 10^–03^	0.62	–4.26
UBQLN1	Q9UMX0	3.2	4.86 × 10^–05^	3.42 × 10^–02^	3.51 × 10^–04^	0.13	1.4	3.94 × 10^–02^	5.84 × 10^–02^	0.11	–0.32

a These 12 proteins also show a significant correlation with increased patient survival if down regulated in Lymph node metastasis. FC: fold change, HR: hazard ratio, K–W P.adj: Adjusted p value of Kruskall–Wallis test.

In contrast, the elevated expression of the remaining 33 proteins was positively associated with favorable patient outcomes. These proteins were involved in diverse biological processes but showed significant enrichment within specific cellular compartments, particularly around the ribosome (Figure 5). For a more detailed perspective, Figure 6 offers a comprehensive overview of the expression patterns of all survival-associated key proteins across all samples.

**Figure 5:**
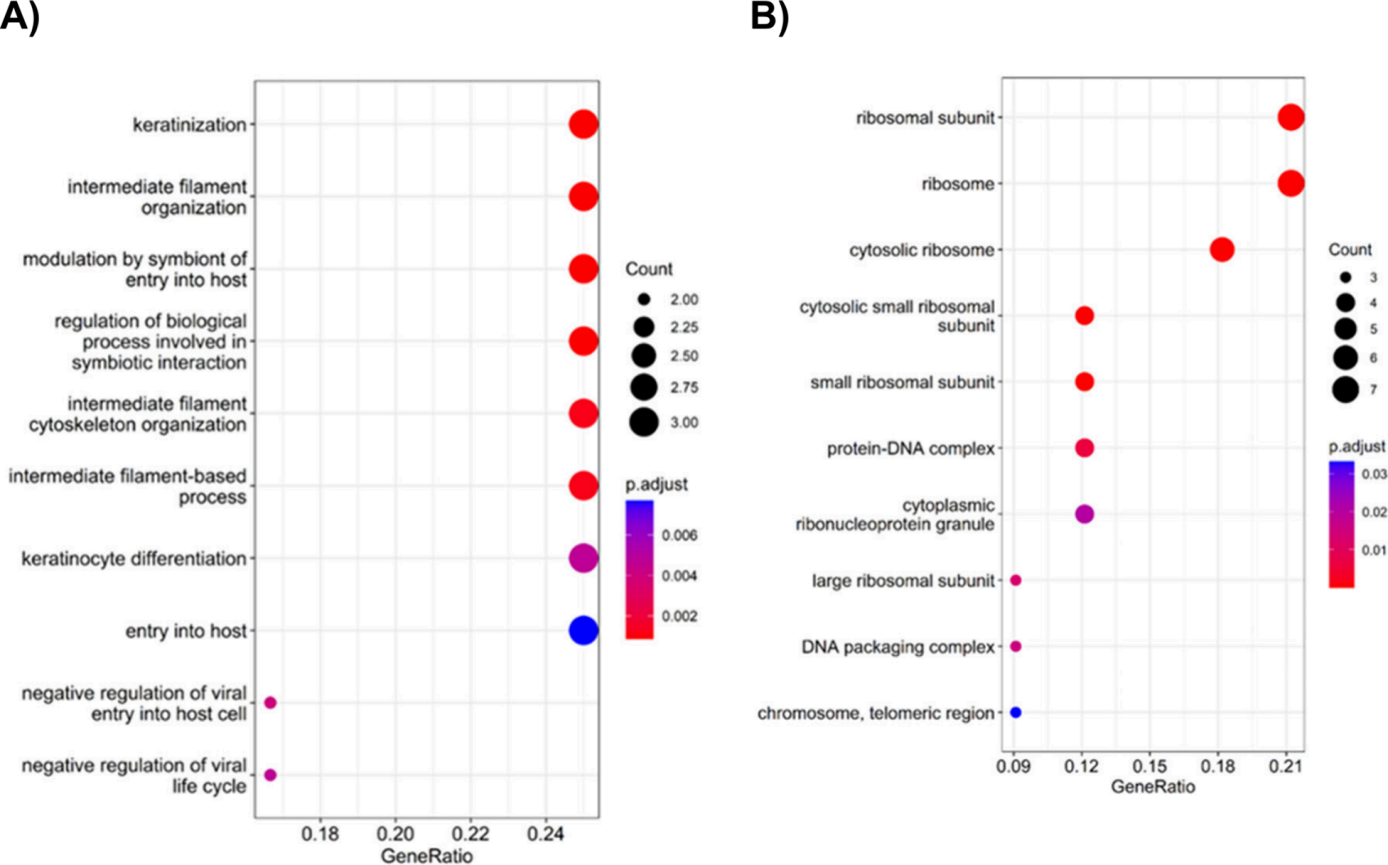
Functional enrichment analysis of the 12 proteins related to longer survival if downregulated (A) and 33 survival-related proteins with favorable prognosis if upregulated in metastatic lymph nodes (B).

**Figure 6:**
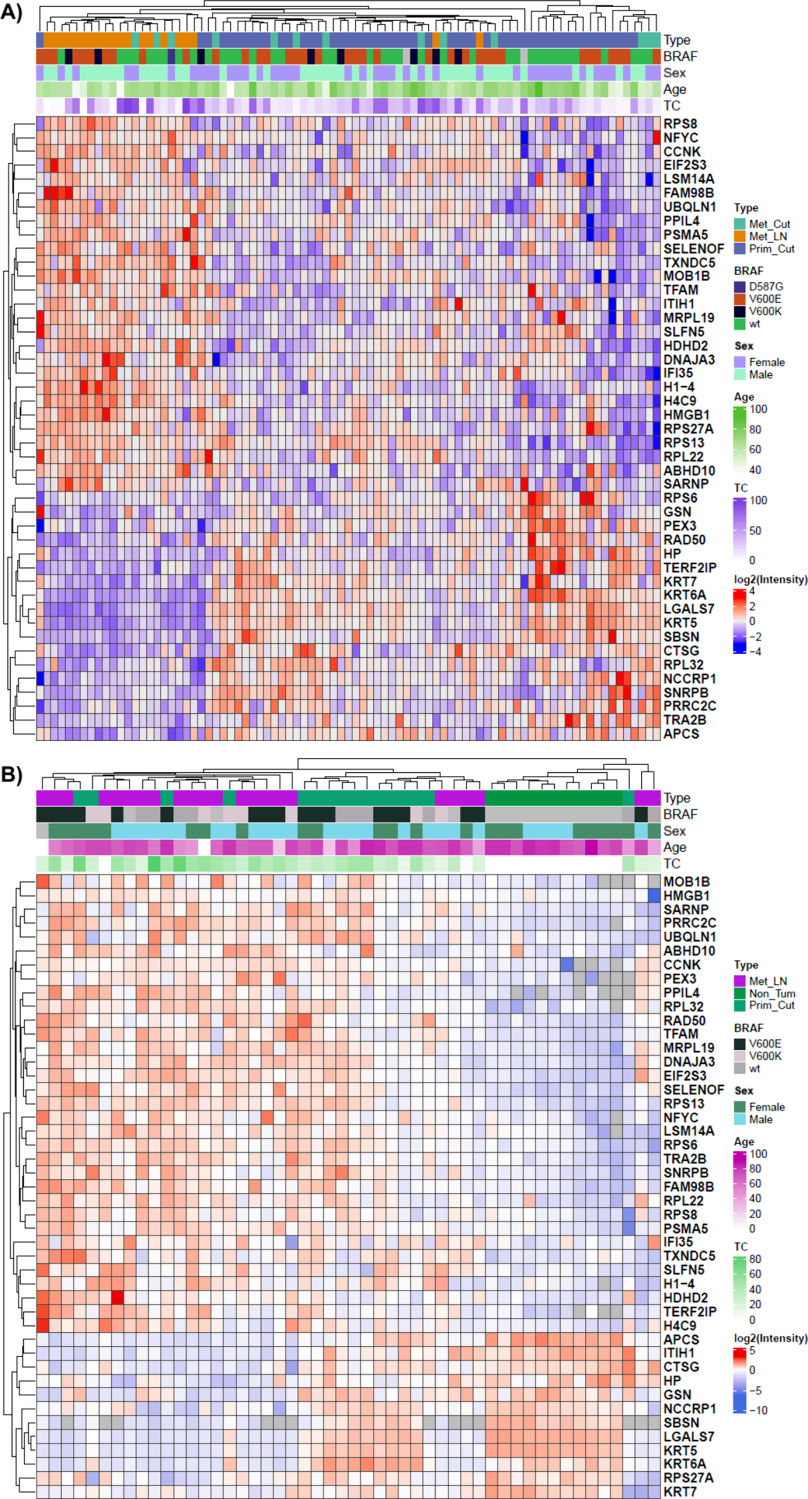
Heatmap representation of the expression of the 45 proteins correlated with different survival outcomes in the Cero (A) and Cuarto (B) cohorts. TC: Tumor content, Met_Cut: Cutaneous metastasis, Met_LN: Lymph node metastasis, Prim_Cut: Primary melanoma.

## Discussion

Our study was driven by the aim to create a user-friendly tool that provides access to data from different melanoma cohorts. To accomplish this, we collected tumor samples from five distinct cohorts, each containing a substantial number of specimens. Subsequently, we crafted a web portal by which users can easily create publication-ready visualizations and access the database. Through this platform, we executed a comprehensive analysis pipeline with the intent of pinpointing a curated array of potential biomarker proteins related to melanoma progression.

The established web platform enables unrestricted exploration of all cohorts included in our database. The *exploratory analysis* option offers an expression profile of a selected protein, providing users with a comprehensive outline of proteins expressed in multiple tissue types and studies. The exploratory analysis feature provides users with a quick overview of our database, acting as a starting point for more in-depth searches on specialized pages. With this panel our aim was to offer users an initial insight into their selected protein of interest, which can serve as a foundation for further, more detailed analyses. Additionally, the *group comparison* feature visually presents protein expression profiles based on two clinical variables, using either violin plots or scatterplots for clarity. The *survival analysis* feature estimates the impact of a chosen protein’s expression on overall survival in the studies with available survival data. Furthermore, one can apply histopathologic filters, such as tumor content for additional refinement. The generated output includes a Kaplan–Meier plot, a number-at-risk table, and a summary encompassing p-values, hazard ratios, and median survival in high and low expression cohorts. The *correlation analysis* feature examines two selected proteins using statistical tests like Pearson and Spearman tests. Another valuable feature is the comparison of a selected protein with all available proteins. It compiles a list of proteins with correlation coefficients exceeding or falling below a predefined threshold interval. These capabilities empower researchers, even those lacking extensive bioinformatics expertise, to conduct comprehensive proteomic data analysis. We trust that our webpage exemplifies our dedication to ensuring the accessibility of anonymized patient data, thereby safeguarding the confidentiality and integrity of data in line with the BBMRI ethical principles and governance principles (for more details see https://www.bbmri-eric.eu/wp-content/uploads/AoM_10_8_Access-Policy_FINAL_EU.pdf).

Among the prominent candidates arising from the pool of differentially expressed proteins, several notable contenders come to the fore, notably HP, LGALS7, and UBQLN1. HP, renowned for its multifaceted functionality, plays a dual role. It serves to bind free plasma hemoglobin, thus facilitating the access of degradative enzymes to hemoglobin, while concurrently preventing the loss of iron through the kidneys. Recent research has highlighted haptoglobin’s potential role as a marker for melanoma progression.^25^ Galectin 7 (LGALS7), a crucial member within the beta-galactose-binding protein family, plays a crucial part in cell-to-cell interactions as well as interactions with the extracellular matrix. Notably it is expressed by keratinocytes, alterations in its expression may transpire during the course of carcinogenesis.^26^ UBQLN1 (ubiquilin-1), forms associations with ubiquitin complexes, thereby facilitating their coupling to the proteasome for degradation. While the precise involvement of UBQLN1’s in the progression of cancer remains to be fully elucidated, some studies tentatively suggest its potential role in suppressing metastasis particularly within the context of lung cancer.^27^ It is essential to acknowledge the study’s limitations to fully appreciate the platform’s capabilities. Heterogeneous clinical data and variances in quantitative protein measurement methods across the five cohorts posed challenges for seamless data integration. The distinction between label-free and TMT-based quantification methods introduces relative quantification results on different scales,^28^ making batch correction challenging. We tackled this issue by implementing a modular design, each tailored to a specific original data set, thereby overcoming the issue of methodological inconsistencies. Notably, not all patients had complete clinical data, which may restrict the applicability of our platform in specific patient subcohorts. As of our knowledge MEL-PLOT stands as the only protein-based analysis tool specifically tailored for melanoma, providing a unique opportunity to explore melanoma-specific proteins using various patient characteristics across normal, primary, and metastatic melanoma samples.

In conclusion, the MEL-PLOT platform serves as an exploratory tool in melanoma proteomics. A pivotal strength of this platform is its incorporation of a substantial number of cases with available clinical follow-up, including survival data. We plan to add additional patient cohorts as they become available and keep the platform up-to-date and constantly update the platform according to user inputs. It is not merely a data repository but a powerful analytical tool that democratizes access to complex proteomic data. We envision our platform to be a model example of making patient data both accessible and actionable for the broader research community, thereby accelerating the pace of melanoma research and potentially leading to improved patient outcomes.

## Supplementary Material









## Data Availability

The data sets generated and/or analyzed during this study, along with the associated scripts, are available at the following link: https://github.com/4ronB/MEL-PLOT. Additionally, the raw mass spectrometry proteomics data have been deposited in the ProteomeXchange consortium via the PRIDE partner repository with the data set identifiers PXD028930, PXD009630, PXD035206, PXD058546, and PXD026086.

## Notes

Figure S1: Upset plot illustrating the quantified proteins in each cohort and their overlaps (PDF)
Table S1: Mapping table listing the UniProt IDs assigned to the cohorts (XLSX)
Table S2: Global results of differential protein expression analysis in Cero (XLSX)
Table S3: Global results of differential protein expression analysis in Cuarto (XLSX)
