# Supplementary material for: Melanoma Proteomics Unveiled: Harmonizing Diverse Data Sets for Biomarker Discovery and Clinical Insights via MEL-PLOT
Source: J Proteome Res. 2025 May 5;24(6):3117–28. doi: 10.1021/acs.jproteome.4c00749 (PMC12150306; doi:10.1021/acs.jproteome.4c00749)
Supplement: Supplementary file 1 [file pr4c00749_si_001.pdf]

Supporting information

Melanoma Proteomics Unveiled: Harmonizing Diverse Datasets for Biomarker Discovery and Clinical Insights via MEL-PLOT

Áron Bartha[1, 2], Boglárka Weltz[1, 3], Lazaro Hiram Betancourt[4, 5], Jeovanis Gil[4, 5], Natália Pinto de Almeida[4, 5], Giampaolo Bianchini[6], Beáta Szeitz[7], Leticia Szadai[8], Indira Pla [9, 4], Lajos Kemény[10, 11, 12], Ágnes Judit Jánosi[8], Runyu Hong[13, 14], Ahmad Rajeh[15], Fábio Nogueira[16], Viktória Doma[12], Nicole Woldmar[17, 4], Jéssica Guedes[4, 5, 17], Zsuzsanna Újfaludi[18], Yonghyo Kim[19], Tibor Szarvas[20, 21], Zoltan Pahi[22, 23], Tibor Pankotai[22, 24, 23], A. Marcell Szasz[25], Aniel Sanchez[26], Bo Baldetorp[27], József Tímár[28], István Balázs Németh[8], Sarolta Kárpáti[12], Roger Appelqvist[5], Gilberto Barbosa Domont[16], Krzysztof Pawlowski[26, 29, 30], Elisabet Wieslander[26], Johan Malm[31], David Fenyo[13, 14], Peter Horvatovich[32], György Marko-Varga[4, 5], Balázs Győrffy*[1, 33, 3]

[1]Department of Bioinformatics, Semmelweis University, Budapest 1085, Hungary
[2]Department of Pediatrics, Semmelweis University, Budapest 1085, Hungary
[3]Cancer Biomarker Research Group, Institute of Molecular Life Sciences, Research Centre for Natural Sciences, H-1117, Budapest, Hungary
[4]European Cancer Moonshot Lund Center, SE-221 84 Sweden
[5]Clinical Protein Science & Imaging, Biomedical Centre, Department of Biomedical Engineering, Lund University, Lund,223 63, Sweden
[6]San Raffaele Hospital, Milan, 20132, Italy
[7]Division of Oncology, Department of Internal Medicine and Oncology, Semmelweis University, Budapest,1085 Hungary
[8]Department of Dermatology and Allergology, University of Szeged, Szeged,6720, Hungary
[9]Department of Biomedical Engineering, Faculty of Engineering, LTH, Lund University, Lund, 22363 Sweden
[10]HCEMM-SU Translational Dermatology Research Group, Semmelweis University, Budapest,1085, Hungary
[11]Department of Physiology, Semmelweis University, Budapest,1094, Hungary
[12]Department of Dermatology, Venerology and Dermatooncology, Faculty of Medicine, Semmelweis University, Budapest, 1085,Hungary
[13]Institute for Systems Genetics, NYU Grossman School of Medicine, New York, NY 10016, USA
[14]Department of Biochemistry and Molecular Pharmacology, NYU Grossman School of Medicine, New York, NY 10016, USA
[15]Department of Dermatology, Massachusetts General Hospital, Harvard Medical School, Boston,02114, MA, USA
[16]Proteomics Unit, Institute of Chemistry and Research Center for Precision Medicine, Institute of Biophysics Carlos Chagas Filho, Federal Univesity of Rio de Janeiro, Rio de Janeiro, 21941-170 Brazil
[17]Chemistry Institute Federal, University of Rio de Janeiro, Rio de Janiero, 21941-909 Brazil
[18]University of Szeged, Albert Szent-Györgyi Clinical Centre, Department of Pathology, 6720, Szeged, Hungary
[19]Drug Discovery Platform Research Center, Therapeutics and Biotechnology Division, Korea Research Institute of Chemical Technology, Daejeon, 34114, Republic of Korea
[20]Department of Urology, Semmelweis University, Budapest,1082, Hungary
[21]Department of Urology, University of Duisburg-Essen,45147, Germany
[22]Department of Pathology, Albert Szent-Györgyi Medical School, University of Szeged, Állomás utca 1, Szeged H-6725, Hungary
[23]Hungarian Centre of Excellence for Molecular Medicine (HCEMM), Genome Integrity and DNA Repair Core Group, University of Szeged, Budapesti út 9, Szeged H-6728, Hungary
[24]Competence Centre of the Life Sciences Cluster of the Centre of Excellence for Interdisciplinary Research, Development and Innovation, University of Szeged, Dugonics tér 13, Szeged H-6720, Hungary
[25]Division of Oncology, Department of Internal Medicine and Oncology, Semmelweis University, 1085 Budapest, Hungary
[26]Section for Clinical Chemistry, Department of Translational Medicine, Lund University, Skåne University Hospital

Malmö, Malmö,205 02, Sweden
[27]Division of Oncology, Department of Clinical Sciences Lund, Lund University, Lund,221 84, Sweden
[28]Department of Pathology, Forensic and Insurance Medicine, Faculty of Medicine, Semmelweis University, Budapest,1085, Hungary.
[29]Department of Biochemistry and Microbiology, Warsaw University of Life Sciences, Warszawa,02-787, Poland
[30]Department of Molecular Biology, University of Texas Southwestern Medical Center, 75390-9148, Texas, USA
[31]Section for Clinical Chemistry, Department of Translational Medicine, Lund University,21428 Sweden
[32]University of Groningen, Groningen Research Institute of Pharmacy, Analytical Biochemistry, Groningen,9711, The Netherlands
[33]Dept. of Biophysics, Medical School, University of Pecs, H-7624, Pecs, Hungary

Table of contents

Table S1 - Mapping table listing the UniProt IDs assigned to the cohorts

Table S2 - Global results of differential protein expression analysis in Cero

Table S3 - Global results of differential protein expression analysis in Cuarto

Figure S1 - Upset plot illustrating the quantified proteins in each cohort and their overlaps

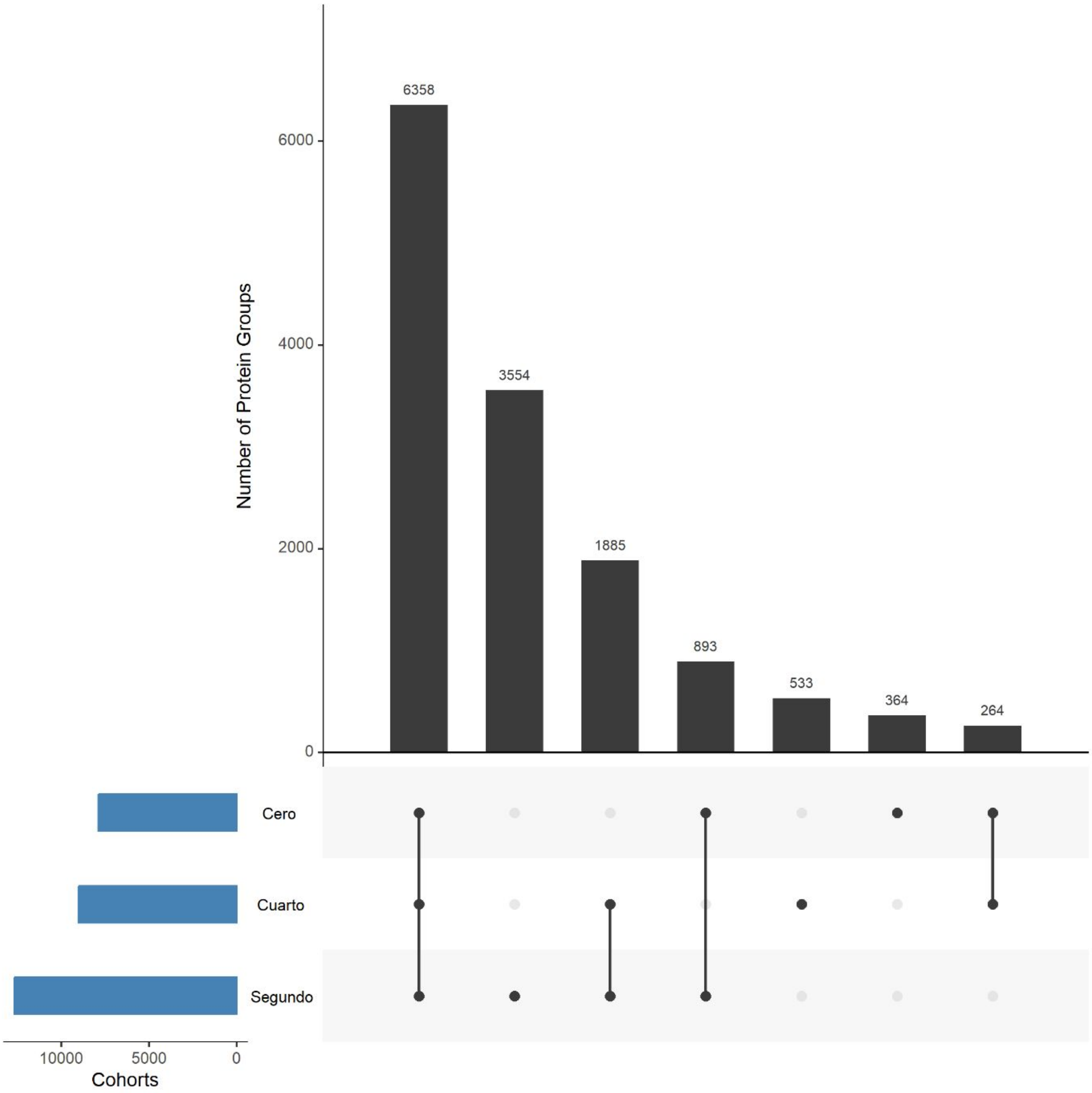

Figure S1: upset plot showing the quantified proteins in Cero, Cuarto and Segundo cohorts and their overlaps
